# Use of a colorimetric (DELI) test for the evaluation of chemoresistance of *Plasmodium falciparum* and *Plasmodium vivax* to commonly used anti-plasmodial drugs in the Brazilian Amazon

**DOI:** 10.1186/1475-2875-12-281

**Published:** 2013-08-12

**Authors:** Lilian R Pratt-Riccio, Yonne F Chehuan, Maria José Siqueira, Maria das Graças Alecrim, Cesare Bianco-Junior, Pierre Druilhe, Philippe Brasseur, Maria de Fátima Ferreira-da-Cruz, Leonardo JM Carvalho, Cláudio T Daniel-Ribeiro

**Affiliations:** 1 Laboratório de Pesquisas em Malária, Instituto Oswaldo Cruz, Fiocruz, Avenida Brasil 4365, Manguinhos, Rio de Janeiro, RJ, Brazil CEP: 21040-900; 2 Centro de Pesquisa, Diagnóstico e Treinamento em Malária (CPD-Mal), Fiocruz, Reference Center for Malaria in the Extra-Amazonian Region for the Secretary for Health Surveillance from the Ministry of Health, Rio de Janeiro, RJ, Brazil; 3 Laboratório de Malária, Fundação de Medicina Tropical Dr Heitor Vieira Dourado, Manaus, Amazonas, Brazil; 4 Institut Pasteur, Paris, France; 5 UMR 198, Institut de Recherche pour le Développement, Dakar, Sénégal, Bangladesh

**Keywords:** Malaria, *Plasmodium falciparum*, *Plasmodium vivax*, Resistance, Anti-malarials, DELI test

## Abstract

**Background:**

The emergence and spread of *Plasmodium falciparum* and *Plasmodium vivax* resistance to available anti-malarial drugs represents a major drawback in the control of malaria and its associated morbidity and mortality. The aim of this study was to evaluate the chemoresistance profile of *P. falciparum* and *P. vivax* to commonly used anti-plasmodial drugs in a malaria-endemic area in the Brazilian Amazon.

**Methods:**

The study was carried out in Manaus (Amazonas state), in the Brazilian Amazon. A total of 88 *P. falciparum* and 178 *P. vivax* isolates was collected from 2004 to 2007. The sensitivity of *P. falciparum* isolates was determined to chloroquine, quinine, mefloquine and artesunate and the sensitivity of *P. vivax* isolates was determined to chloroquine and mefloquine, by using the colorimetric DELI test.

**Results:**

As expected, a high prevalence of *P. falciparum* isolates resistant to chloroquine (78.1%) was observed. The prevalence of isolates with profile of resistance or decreased sensitivity for quinine, mefloquine and artesunate was 12.7, 21.2 and 11.7%, respectively. In the case of *P. vivax*, the prevalence of isolates with profile of resistance for chloroquine and mefloquine was 9.8 and 28%, respectively. No differences in the frequencies of isolates with profile of resistance or geometric mean IC50s were seen when comparing the data obtained in 2004, 2005, 2006 and 2007, for all tested anti-malarials.

**Conclusions:**

The great majority of *P. falciparum* isolates in the Brazilian malaria-endemic area remain resistant to chloroquine, and the decreased sensitivity to quinine, mefloquine and artesunate observed in 10–20% of the isolates must be taken with concern, especially for artesunate. *Plasmodium vivax* isolates also showed a significant proportion of isolates with decreased sensitivity to chloroquine (first-line drug) and mainly to mefloquine. The data presented here also confirm the usefulness of the DELI test to generate results able to impact on public health policies.

## Background

Malaria is one of the most serious public health problems worldwide. It is a leading cause of disease and death in many developing countries, where young children and pregnant women are the most affected groups. Malaria is endemic in 99 countries and, according to the World Health Organization (WHO), there were about 219 million cases of malaria and an estimated 660,000 malaria deaths in 2010 [1].

In Brazil, malaria is hypo- to meso-endemic, present throughout the year with clear seasonal fluctuations and is frequently associated with migration movements of non-immune individuals to areas where malaria is endemic [2]. In 2011, approximately 263,000 malaria cases were reported in Brazil, 99.8% of them in the Amazon Region [3].

In the absence of an effective vaccine, malaria control is almost entirely dependent on the availability of effective chemotherapy and the efficacy of anti-vectorial measures. The emergence and spread of *Plasmodium falciparum* resistance to anti-malarial drugs thus represent a major drawback in the control of malaria and its associated morbidity and mortality. Resistance to current available anti-malarial drugs has been reported in many places in the world, including Brazil. The case of chloroquine is representative. Chloroquine appeared in the 1940s as an extraordinary anti-malarial drug: effective, cheap and easy to produce. However, since the first reports of *P. falciparum* parasites with reduced sensitivity to chloroquine [4-7], the resistance to this drug spread rapidly throughout the world. In many places, it became no longer effective and had to be substituted by other drugs, more expensive and in many cases presenting considerable side effects. The same was true in Brazil, where the first report of *P. falciparum* resistance to chloroquine was in the 1960s [4] and, by 1990, more than 95% of *P. falciparum* isolates circulating in malaria endemic areas were resistant to chloroquine [8].

Given the strong impact of chemoresistance to anti-malarial drugs in the control of malaria, monitoring the development of resistant phenotypes is a priority wherever malaria is endemic. The *in vitro* methods for evaluating anti-malarial drug sensitivity can provide a profile of plasmodial sensitivity to a variety of drugs, since several drugs can be assayed simultaneously. A lower sensitivity *in vitro* of an isolate does not necessarily mean that it will be resistant *in vivo,* because the *in vivo* outcome depends on a number of factors that cannot be evaluated *in vitro*. However, it can provide an outline of resistant phenotypes circulating for each drug tested when using samples from an adequate number of patients in a given area, thus providing crucial information for policy-makers to determine the best drug to be employed in the area. Ideally, monitoring of antimalarial chemoresistance must be continuous, since development and spread of resistance are dynamic events, changing with time and according to human interventions and other factors, such as population migration.

A good alternative for microscopic and isotopic *in vitro* methods appeared with the development of immuno-enzymatic methods to evaluate plasmodial growth. The double-site enzyme-linked immunodetection (DELI) test [9,10] is based on the detection of the parasite lactate dehydrogenase (LDH), produced during the plasmodial growth, by means of monoclonal antibodies, in a sandwich ELISA. Deli-test assay proved to be reproducible, very sensitive to detect low parasitaemia (0.005%) and easy to perform even in field conditions, and without the need of well training microscopists or manipulation of [3] hypoxanthine [10-13]. As an ELISA procedure, it allows the fast evaluation of many samples at once. In addition, DELI-test is easier to perform, faster to implement, and cheaper than *in vitro* isotopic assays [10]. With the development of an efficient short-term culture protocol for *in vitro Plasmodium vivax* culture [14], and the availability of monoclonal antibodies specific for *P.vivax* LDH, DELI test also allows the study of the sensitivity of this parasite to anti-malarial drugs.

The aim of this study was to evaluate the chemoresistance profile of *P. falciparum* and *P. vivax* to commonly used anti-plasmodial drugs in a Brazilian malaria-endemic area in the Amazon Region using the DELI-test.

## Methods

### Studied area and isolates

This study was carried out in the city of Manaus (Amazonas state), located at the Brazilian Amazon. A total of 88 *P. falciparum* and 178 *P. vivax* isolates were collected from patients who sought health care at Dr Heitor Vieira Dourado Tropical Medicine Foundation (FMT-HVD) from 2004 to 2007.

Written informed consent was obtained from all donors and venous blood samples were drawn in Vacutainer® (Becton Dickinson, Oxnard, CA) EDTA tubes. Thin and thick blood smears were examined for identification of malaria parasite and determination of parasitaemia by two expert malaria microscopists from FMT-HVD and from the Laboratory of Malaria Research (Fiocruz, Rio de Janeiro, Brazil) which is a reference centre in malaria diagnosis for the Brazilian Ministry of Health. Thick blood smears from all subjects were stained with Giemsa and examined under 1,000-fold magnification. Parasitaemia was determined by counting parasites in reference to 200 white blood cells in thick blood films, and the number of the blood parasites per millilitre was calculated.

All malaria patients enrolled in this study complied with the following criteria: 1) they presented symptoms; 2) they had *P. falciparum* or *P. vivax* infection; 3) they neither used chemoprophylaxis nor took anti-malarial drugs as self-treatment; 4) they were 12 years old or older; 5) females were not pregnant or breast feeding; and, 6) blood collection was performed at the day of diagnosis before malaria treatment. After malaria diagnosis and blood sample collection, the patients were immediately treated according to the Brazilian Ministry of Health standards for malaria therapy.

The study was reviewed and approved by the Fundação Oswaldo Cruz Ethical Committee (number 221/03).

### Drug sensitivity assay

The sensitivity of *P. falciparum* isolates was determined to chloroquine sulphate, quinine hydrochloride, mefloquine hydrochloride, and artesunate and the sensitivity of *P. vivax* isolates was determined to chloroquine sulphate and mefloquine hydrochloride (all drugs obtained from Sigma-Aldrich).

Anti-malarial drugs aliquoted in predosed tubes (15 mg/tube) were dissolved by the addition of 3 mL of 100% ethanol and 7 mL of RPMI-1640 medium (Gibco, Invitrogen Life Technologies) (chloroquine and quinine) or 10 mL of 100% methanol (mefloquine and artesunate). From the stock solution, another solution was prepared for each drug at final concentrations of 2,400 ng/mL for chloroquine, 1,200 ng/mL for quinine and 150 ng/mL for mefloquine and artesunate in RPMI-1640 medium supplemented with 0.5% albumax (Gibco) (complete culture medium), for *P. falciparum*; and 600 ng/mL for chloroquine and 300 ng/mL for mefloquine in a 3:1 mix (vol/vol) of RPMI 1640 medium and Waymouth medium (Sigma-Aldrich), for *P. vivax.* One hundred mL of each dilution were added to wells of the column 1 of 96-well tissue culture plates (Falcon) and nine subsequent two-fold dilutions were prepared in wells 2–9. Wells 10–12 were filled with 50 μL of complete culture medium (culture control wells). Each concentration of anti-malarial was tested in duplicate (*P. falciparum*) or quadruplicate (*P. vivax*).

Isolates with at least 0.1% parasitaemia were included in the study and were maintained at 4°C (for up to 8 hr after collection) before the *in vitro* culture was started. Blood samples were washed twice with a solution of RPMI-1640 medium and then resuspended in the complete culture medium (*P. falciparum*) or RPMI-Waymouth (*P. vivax*). If necessary, a dilution was performed by adding uninfected O-positive-group erythrocytes to obtain a 0.5–1% parasite density. Finally, 200 μL of this suspension were distributed to each well in the anti-malarial predosed plates in 1.2% final haematocrit. The plates were incubated for 48 hr at 37°C in a CO_2_ incubator (5% CO_2_ in air) and were then frozen and kept at −20°C. Before ELISA, the plates were frozen and thawed three times to lyse the red blood cells.

### ELISA

The success of the drug sensitivity assay and the appropriate volume of haemolysed culture were previously determined for each clinical isolate by using a preliminary LDH ELISA as a pretest. To determine which dilution of haemolysed culture had to be used in the DELI-test, four serial dilutions (1:50, 1:25, 1:12.5, 1:6.25) of culture control wells (no drug) of each isolate were tested in a preliminary LDH ELISA. The dilutions used were those providing optical density (OD) readings between 1.0–2.0.

ELISA plate (Nunc, Maxisorb, Denmark) wells were coated with 100 μL of monoclonal antibody (MAb) against *P. falciparum* (17EA) or *P. vivax* (11D) LDH (kind gifts of Dr Michael Makler, Flow Inc, Portland, USA) at 1 μg/mL, in PBS pH 7.4. The plates were incubated overnight at 4°C, washed with PBS with 1% BSA (fraction V, Boehringer-Mannheim) (PBS-BSA) and then incubated with 300 μL of PBS-BSA for 4 hr, at room temperature. The plates were maintained at 4°C until used.

Subsequently, the appropriated volume of the haemolysed culture was transferred to the ELISA plate wells with PBS-BSA to the final volume of 100 μL, incubated for 1 hr at 37°C and then washed with PBS-BSA. After the addition of 100 μL per well of a biotinylated MAb against pan-*Plasmodium* LDH (19G7), the plates were incubated for 1 hr at 37°C. After washing, a third incubation for 30 min at room temperature with 100 μL of a streptavidin horseradish peroxidase solution was followed by a last washing step. Enzyme activity was revealed by incubation for 5 min at room temperature with 100 μL of tetramethylbenzidine (TMB). The reaction was stopped with 1 M of phosphoric acid, and the absorbance was read at 450 nm in a spectrophotometer (Spectramax 250, Molecular Devices).

Concentration-response data were analysed by a non-linear regression function to determine the 50% inhibition of parasite growth (IC50), defined as the concentration of the drug which inhibited 50% of the production of LDH as determined by OD values from sample test wells compared to those obtained from drug-free control wells. The IC50 threshold values for resistance to chloroquine, mefloquine, quinine and artesunate were 100 nM, 30 nM, 500 nM and 10 nM, respectively; these values were similar to those already described elsewhere [10,11,15,16].

### Statistical analysis

The data were stored in the Fox-plus® (Borland International Inc, Perrysburg, OH, USA) data bank software. GraphPad Instat and GraphPad Prism (GraphPad Software Inc, CA, USA) statistical software programs were used for data analysis. The Student’s t-test was used to analyse the differences in IC50 mean values and the Chi-square test was applied to compare the prevalence of isolates with profile of resistance observed from 2004 to 2007.

## Results

A total of 88 *P. falciparum* and 178 *P. vivax* samples was collected from malaria patients from 2004 to 2007. Males were predominant in both *P. falciparum* (71%) and *P. vivax* (81%) groups. The average age was also similar between *P. falciparum* (36 ± 15 years old) and *P. vivax* (34 ± 13 years old) groups.

**Figure 1:**
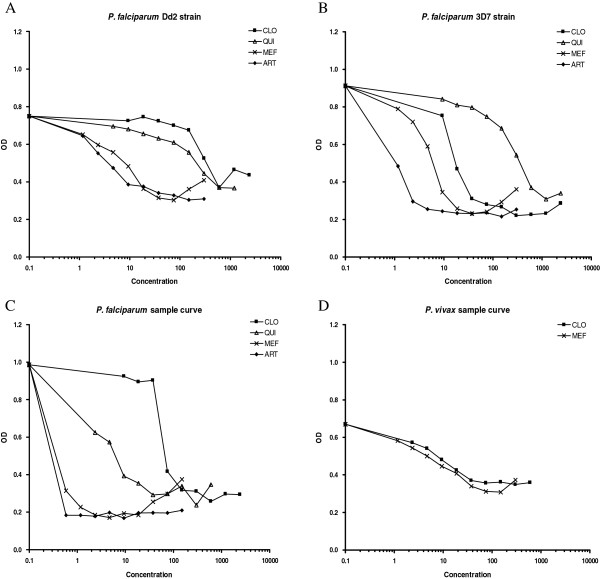
**Example of curves for *Plasmodium falciparum *and *Plasmodium vivax. ****P. falciparum* chloroquine-resistant Dd2 strain **(A)**, *P. falciparum* chloroquine-sensitive 3D7 strain **(B)**, isolate from one patient with *P. falciparum* infection **(C)** and isolate from one patient with *P. vivax* infection **(D)** cultures in presence of chloroquine, quinine, mefloquine and artesunate (*P. falciparum*) or chloroquine and mefloquine (*P. vivax*).

Overall, the IC50 values could be determined in most of *P. falciparum* (70/88, 80%) and *P. vivax* (132/178, 74%) samples (Table 1). Successful *in vitro* culture allowing IC50 determination for fresh isolates was low in the first year (2004) for both *P. falciparum* and *P. vivax* cultures, but substantially increased in the subsequent years, suggesting better handling and optimization of culture procedures (Table 1). The feasibility of the procedure using chloroquine-sensitive (Dd2) and chloroquine-resistant (3D7) strains adapted for *in vitro* culture was determined and showed that it worked well (Figure 1).

**Table 1:** **Frequencies of *Plasmodium falciparum *** **and *Plasmodium vivax *** **field isolates from Manaus, Amazonas State, Brazil, with IC50 of commonly used anti-malarial drugs determined in 2004, 2005, 2006 and 2007**

	**2004 nd/n (%)**	**2005 nd/n (%)**	**2006 nd/n (%)**	**2007 nd/n (%)**	**Total nd/n (%)**
***P. falciparum***	16/30 (53)	18/19 (94)	20/22 (91)	16/17 (94)	70/88 (80)
***P. vivax***	11/23 (48)	24/44 (55)	32/38(84)	65/73(89)	132/178 (74)

nd: number of samples with IC50 determined; n: number of total samples (percentage).

**Figure 2:**
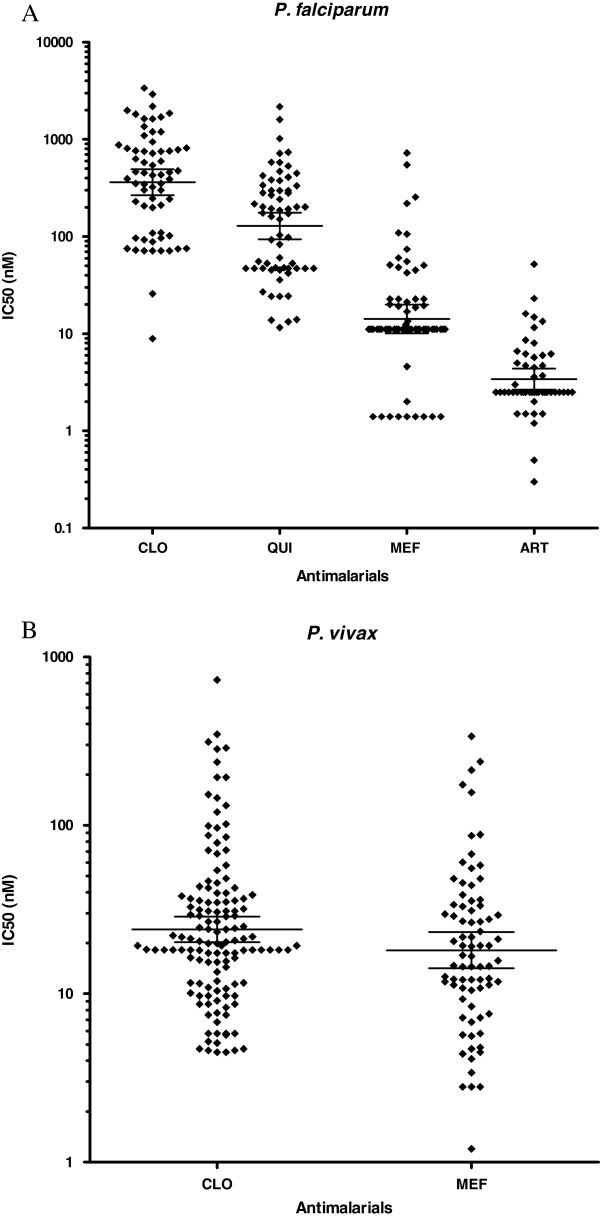
**Distribution of IC50 values in *Plasmodium falciparum *and *Plasmodium vivax *isolates using DELI test. ***Plasmodium falciparum* isolates **(A)**, *Plasmodium vivax* isolates **(B)**. Values are individual IC50 (nM). Lines represent geometric mean with 95% confidence interval.

As expected, a high prevalence of *P. falciparum* isolates resistant or with decreased susceptibility to chloroquine was observed (Figure 2A). Indeed, 78.1% (50/64) of the samples showed IC50 higher than 100 nM. The geometric mean IC50s for chloroquine was 361.8 nM (95% confidence interval 266–492 nM) (Table 2). For quinine, the prevalence of clearly resistant isolates (IC50 > 500 nM) was 12.7% (8/63). The geometric mean IC50s for quinine was 128.5 nM (95% confidence interval 93–175). For mefloquine, the prevalence of isolates with profile of resistance or decreased susceptibility (IC50 > 30nM) was 21.2% (14/66). The geometric mean IC50s for mefloquine was 14.2 nM (95% confidence interval 10–20). Finally, for artesunate, the prevalence of isolates with IC50 above the threshold of 10 nM was 11.7% (6/51). The geometric mean of IC50s was 3.4 nM (95% confidence interval 3–4). No difference in frequencies of isolates with profile of resistance or geometric mean IC50s were seen comparing the data obtained in 2004, 2005, 2006 and 2007 for chloroquine, quinine, mefloquine and artesunate.

**Table 2:** **IC50 values of commonly used anti-malarial drugs in *in vitro *** **culture of *Plasmodium falciparum *** **and in *Plasmodium vivax *** **from Manaus, Amazonas State, Brazil**

	***P. falciparum***				***P. vivax***			
		**IC50 (nM)**				**IC50 (nM)**		
**Drug**^**1**^	**No. of isolates**	**Geometric mean**	**95% CI**^**2**^	**Range**	**No of isolates**	**Geometric mean**	**95% CI**^**2**^	**Range**
Chloroquine	64	361.8	266–492	9–3373	132	24.1	20–29	5–729
Quinine^1^	63	128.5	93–175	12–2174	-	-	-	-
Mefloquine	66	14.2	10–20	2–723	78	18.1	14–23	2–337
Artesunate^1^	51	3.4	3–4	0.3–52	-	--	--	-

^1^Performed only in *P. falciparum* cultures; ^2^CI, confidence interval.

Among *P. falciparum* isolates, different chloroquine, quinine, mefloquine, and artesunate sensitivity levels were observed, however, the temporal analysis indicate that the profile of sensitivity to these drugs remained relatively stable.

In the case of *P. vivax* isolates, the prevalence of isolates with IC50 above threshold of 100 nM for chloroquine was 9.8% (13/132) (Figure 2B) and the geometric mean IC50 was 24.1 nM (95% confidence interval 20–29). For mefloquine, the prevalence of IC50 above threshold of 30 nM was 28% (22/78) and the geometric mean IC50 was 18.1 nM (95% confidence interval 14–23) (Table 2). No difference in frequencies of isolates with profile of resistance or geometric mean IC50s were seen comparing the data obtained in 2004, 2005, 2006 and 2007 for chloroquine and mefloquine.

## Discussion

In the present study, the DELI-test was used to determine the profile of *in vitro* sensitivity of *P. falciparum* and *P. vivax* field isolates in Manaus, in the Brazilian Amazon. Despite some initial technical difficulties in the beginning of the study (2004), the performance of the test in 2005–2007 was similar to that observed in previous studies in Africa, which reported test success rates of 81% for *P. falciparum*[13].

Overall, a high prevalence (78.1%) of *P. falciparum* isolates resistant or with decreased sensitivity to chloroquine was observed. This was expected since earlier studies conducted in Amazonia suggested that 100% of the *P. falciparum* isolates circulating in the area were chloroquine-resistant [17]. In addition, most of the “sensitive” isolates had IC50s close to the threshold for sensitivity (100 nM) and therefore represent rather borderline isolates. Only 2/64 (3.1%) of isolates showed clearly low IC50 to chloroquine. Any evidence of the re-emergence of chloroquine-sensitive parasites in Brazil through the documentation of a wild *pfcrt P. falciparum* haplotype (CVMNK) in Amazonian region was already reported [18].

In Brazil, reduced *in vitro* and *in vivo* sensitivity to mefloquine has been reported since 1981 [19-23] and this might explain the 21.2% resistant phenotypes in the present study. In fact, even before the official introduction of mefloquine in Brazilian endemic areas in 1987, *P. falciparum* isolates with profile of resistance to mefloquine had already been reported [24].

In relation to quinine, the prevalence of resistant isolates (IC50 above 500 nM) was 12.7%. Some isolates showed IC50s very close to the threshold. In any case, this scenario is worrisome because a continuous decreasing in parasite sensitivity has been noted in the Brazilian Amazon [17,21,24-27] and quinine plus doxycycline is actually recommended as *P. falciparum* second-line treatment by the Brazilian Malaria Control Programme.

Since 2007 the Brazilian Malaria Control Programme defined artemisinin-based combined therapy (ACT) as the first-line treatment for uncomplicated falciparum malaria. In this study, 11.7% of the isolates showed IC50 above the threshold of 10 nM (11.7%) for artesunate. This is surprising as these samples were collected in a period before the introduction of ACT in the area; therefore, no selection pressure is to be accounted for. A study performed in another area in the Amazonian region in the same period, showed no evidence of decreased sensitivity of local isolates to artesunate or artemether [28]. However, a recent study showed that 23.9% of the studied isolates from Gabon had a reduced susceptibility to dihydroartemisinin [16]. Decreased sensitivity of *P. falciparum* parasites to artemether *in vitro* in French Guiana and Senegal [29] as well as *in vivo* in Cambodia [30] has also been described. Given the immense value of artemisinin derivatives as alternative drugs for multidrug-resistant parasites, authorities must be alert to these data indicating a trend for emergence of isolates with decreased sensitivity to this drug.

The sensitivity of *P. vivax* parasites to anti-malarial drugs has not been widely monitored *in vitro* due the difficulties in cultivating *P. vivax*. Here, the *in vitro* resistance of *P. vivax* fresh isolates to chloroquine and mefloquine was evaluated for the first time in Brazil. Although the threshold of IC50 to define a sample as resistant to chloroquine is not well established for *P. vivax*, it has been proposed that the same threshold used for *P. falciparum* should be used for *P. vivax*[15]. In this case, a considerable frequency (9.8%) of *P. vivax* isolates was above the adopted 100 nM threshold of chloroquine. This frequency was not surprising, because *P. vivax* resistance to chloroquine has been reported in Brazil [31,32], as well as increasing numbers of severe *P. vivax* malaria cases [33,34]. Consequently, the use of DELI test can be an important tool for backing decisions of the Brazilian health authorities in relation to the treatment policy of *P. vivax*.

Mefloquine is usually an alternative treatment for *P. vivax* infections. Interestingly, the prevalence of *P. vivax* isolates with resistant profile to mefloquine was greater (28%) than to *P. falciparum* parasites (21.2%), corroborating a study performed in Thailand where *P. vivax* isolates were more resistant to mefloquine when compared with *P. falciparum* isolates from the same area [35].

It should be highlighted that the *in vivo* outcome depends on a number of factors that cannot be evaluated *in vitro*, including the level of innate and acquired immunity. However*, in vitro* assays act as a preliminary warning system indicating a trend, since *in vitro* resistance may be indicative of clinical resistance.

## Conclusions

The data here presented using DELI test provide important and useful information regarding the sensitivity profile of Brazilian plasmodial isolates to common anti-malarial drugs. For updating and evaluating potential shifts in the profiles of drug sensitivity, periodic such studies associated with the screening using potential molecular markers should be performed.

## Competing interests

The authors declare that they have no competing interests.

## Authors’ contributions

LRPR carried out the DELI test, performed the statistical analysis and drafted the manuscript; CTDR and LJMC conceived the study, participated in its design and coordination and helped to draft the manuscript; MJS and CBJ carried out the DELI test; YFC, MGA, PD, PB and MFFC helped in the design of the study and reviewed the manuscript. All authors have and approved the final manuscript.
